# Crystal structure of ethyl 4-[(*E*)-(4-hy­droxy-3-meth­oxy­benzyl­idene)amino]­benzoate: a *p*-hy­droxy Schiff base

**DOI:** 10.1107/S2056989016008999

**Published:** 2016-06-14

**Authors:** Jing Ling, Padmini Kavuru, Lukasz Wojtas, Keith Chadwick

**Affiliations:** a Department of Industrial and Physical Pharmacy, Purdue University, 575 Stadium Mall Drive, West Lafayette, IN 47907, USA; b Department of Chemistry, University of South Florida, 4202 E Fowler Ave, Tampa, Florida 33620, USA

**Keywords:** crystal structure, *p*-hy­droxy Schiff base, hydrogen bonding

## Abstract

The title *p*-hy­droxy Schiff base, C_17_H_17_NO_4_, was synthesized *via* the condensation reaction of benzocaine with vanillin. The benzyl­idine and benzoate rings are inclined to one another by 24.58 (8)°, and the conformation about the C=N bond is *E*. In the crystal, mol­ecules are linked by O—H⋯N hydrogen bonds, forming zigzag chains propagating along [010]. Adjacent chains are linked by C—H⋯π and weak offset π–π inter­actions [inter­centroid distance = 3.819 (1) Å], forming sheets parallel to (10-2).

## Abstract 2

The title *p*-hy­droxy Schiff base, is the product of a condensation reaction between benzocaine and vanillin. The benzyl­idine and benzoate rings are inclined to one another by 24.58 (8)°, and the conformation about the C=N bond is *E*.

## Chemical context   

The pharmaceutical industry generally seeks to formulate crystalline forms of their active ingredient by their inherent stability (Yadav *et al.*, 2009 ▸; Paul *et al.*, 2005 ▸). Increasing attention is now being paid to crystal engineering for improving crystal properties (Byrn *et al.*, 1999 ▸). One such strategy is co-crystallization due to its potential for enhancing the physicochemical properties of an API, such as solubility, bioavailability, dissolution, and chemical and physical stability (Shan & Zaworotko, 2008 ▸; Good & Rodríguez-Hornedo, 2009 ▸). The term *co-crystal* does not have a clear and consistent definition in the literature (Desiraju, 2003 ▸; Bond, 2007 ▸; Shan & Zaworotko, 2008 ▸). Generally, a co-crystal is defined as *a homogeneous crystalline phase consisting of two or more discrete chemical entities bound together in the crystal lattice through non-covalent, non-ionic mol­ecular inter­actions*.
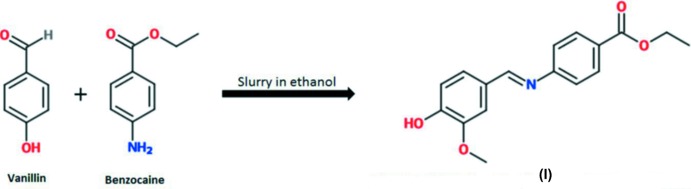


Benzocaine, the ethyl ester of *p*-amino­benzoic acid, is a local anaesthetic which is used to subside pain perception. It relieves pain by inhibiting the voltage-dependent sodium channels on the nerve membrane, which results in stopping the propagation of the action potential. (Neumcke *et al.*, 1981 ▸). In this study, we intended to formulate co-crystals of benzocaine and determine the impact on its physicochemical properties. Vanillin was selected as a potential co-former as it is FDA approved and has the potential to form a strong hydrogen bond between the amine and hy­droxy groups of benzocaine and vanillin, respectively. However, during crystallization a chemical reaction between the two was observed, the product of which is a novel *p*-hy­droxy Schiff base. Schiff bases are an important class of organic compounds with significant bio­logical and chemical importance. In general, they are synthesized by the condensation reaction of an aliphatic or aromatic amine with a carbonyl containing compound, such as an aldehyde, *via* nucleophilic addition. Herein, we report on the crystal structure of the title compound, a new *p*-hy­droxy Schiff base, synthesized from benzocaine and vanillin by slurry crystallization.

## Structural commentary   

**Figure 1:**
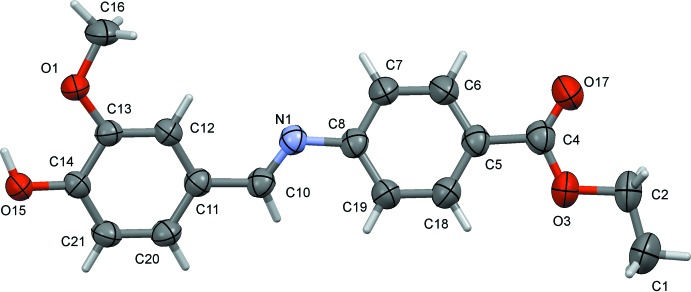
The mol­ecular structure of compound (I), with atom labeling. Displacement ellipsoids are drawn at the 50% probability level.

The title Schiff base, (I), is the product of the reaction of benzocaine with vanillin (Scheme[Chem scheme1]). In the title compound, Fig. 1 ▸, the conformation of the C10=N1 imine bond is *E*. The mol­ecule is non-planar, with a dihedral angle between the aryl rings of 24.58 (8)°. The *m*-meth­oxy group (O1/C13/C16) is slightly out of the plane of the benzene ring (C11–C14/C20/C21) to which it is attached by 5.37 (18)°, while the mean plane of the ethyl­acetate group (O3/O17/C1/C2/C4) is inclined to the benzene ring (C5–C8/C18/C19) to which it is attached by 10.23 (11)°. This non-linearity is consistent for Schiff bases.

## Supra­molecular features   

**Figure 2:**
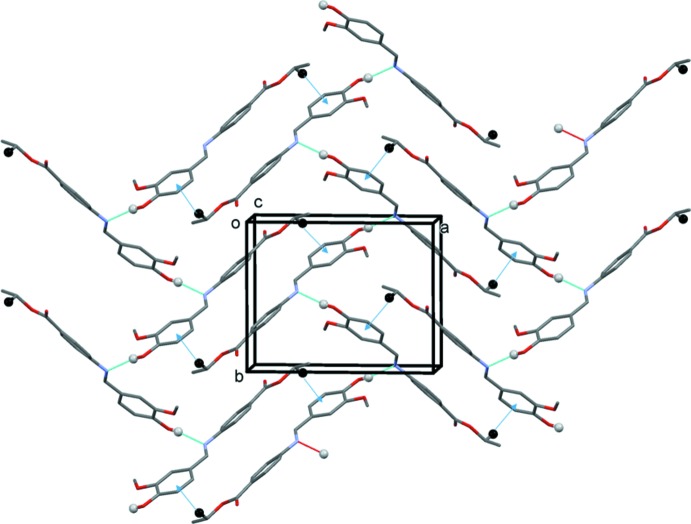
A view along the *c* axis of the crystal packing of compound (I), with hydrogen bonds shown as dashed lines and C—H⋯π inter­actions as blue arrows (see Table 1 ▸).

In the crystal, mol­ecules are linked by O—H⋯N hydrogen bonds, forming zigzag chains propagating along [010]; see Table 1 ▸ and Fig. 2 ▸. Adjacent chains are linked by C—H⋯π inter­actions (Table 1 ▸, Fig. 2 ▸), and weak offset π-π- inter­actions, forming sheets parallel to (10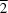) [*Cg*1⋯*Cg*1^i^ = 3.819 (1) Å, inter­planar distance = 3.672 (2) Å, slippage = 1.05 Å, *Cg*1 is the centroid of ring C5–C8/C18/C19; symmetry code: (i) −*x* + 2, −*y* + 1, −*z* + 1],

The crystal structure analysis of compound (I), has shown that, due to the aromatic hy­droxy group being located in the *para* rather than the *ortho* position, this Schiff base cannot form the intra­molecular C=N⋯O—H hydrogen bond responsible for *keto–enol* tautomerism. However, the close proximity of the C=N and O—H groups gives rise to the possibility that external stimulation of the material by heat or light may lead to the zwitterionic form. The potential for compound (I) to form a zwitterionic state, coupled with the non-linear conformation of the mol­ecule in the solid state, suggest that this Schiff base may exhibit inter­esting physical properties, that we are currently in the process of evaluating.

## Database survey   

In the Cambridge Structural Database (CSD, V53.7; Groom *et al.*, 2016 ▸), there are three known Schiff bases synthesized from benzocaine (CSD ref codes: VABSUO; Shakir *et al.*, 2010 ▸, and ZOZROV and ZOZRUB; Kurogoshi & Hori, 1996 ▸), and one derived from vanillin (CSD ref code: LEFVID; Fejfarová *et al.*, 2012 ▸). The dihedral angles between the aryl rings in VABSUO, ZOZROV, ZOZRUB and LEFVID were found to be 24.85 (9), 59.7 (2), 53.94 (9), and 37.87 (10)°, respectively. The N1=C10 and C8—N1 bond lengths of the imine group of the title compound are 1.274 (2) and 1.415 (2) Å, respectively. They are comparable to the imine bond lengths observed for VABSUO, ZOZROV, ZOZRUB and LEFVID, which vary between 1.262 (4)–1.283 (3) Å and 1.414 (7)–1.428 (3) Å, respectively.

## Synthesis and crystallization   

Compound (I) was prepared by slurrying an equimolar mixture of benzocaine (1.16 g, 7 mmol) and vanillin (1.07 g, 7 mmol) in 2 ml of anhydrous ethanol (see Scheme[Chem scheme1]). The slurry was stirred continuously for 18 h at room temperature (296 K). The product was then filtered and air dried before being analysed by powder X-ray diffraction to determine the presence of a new crystalline phase. Single crystals were then prepared by dissolving an equimolar mixture of benzocaine (0.83 g, 5 mmol) and vanillin (0.77 g, 5 mmol) in 10 ml of ethanol. The solution was allowed to evaporate under ambient conditions and yellow block-like crystals were obtained after four days.

## Refinement   

Crystal data, data collection and structure refinement details are summarized in Table 2 ▸. Two H atoms, H15 and H10, were located in a difference Fourier map and freely refined. The remaining H atoms were placed in geometrically calculated positions and included in the refinement process using a riding model: C—H = 0.93–0.97 Å with *U*_iso_(H) = 1.5*U*_eq_(C-meth­yl) and 1.2*U*_eq_(C) for other H atoms.

## Supplementary Material

Crystal structure: contains datablock(s) I, Global. DOI: 10.1107/S2056989016008999/su5304sup1.cif

Structure factors: contains datablock(s) I. DOI: 10.1107/S2056989016008999/su5304Isup2.hkl

Supporting information file. DOI: 10.1107/S2056989016008999/su5304sup3.pdf

Supporting information file. DOI: 10.1107/S2056989016008999/su5304Isup4.cml

CCDC reference: 1483394

Additional supporting information: crystallographic information; 3D view; checkCIF report

## Figures and Tables

**Table 1:** Hydrogen-bond geometry (Å, °) *Cg*2 is the centroid of the C11–C14/C20/C21 ring.

*D*—H⋯*A*	*D*—H	H⋯*A*	*D*⋯*A*	*D*—H⋯*A*
O15—H15⋯N1^i^	0.88 (2)	2.00 (2)	2.828 (2)	156 (2)
C2—H2*B*⋯*Cg*2^ii^	0.97	2.87	3.766 (2)	154

Symmetry codes: (i) 
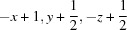
; (ii) 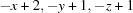.

**Table 2:** Experimental details

Crystal data	
Chemical formula	C_17_H_17_NO_4_
*M*_r_	299.31
Crystal system, space group	Monoclinic, *P*2_1_/*c*
Temperature (K)	296
*a*, *b*, *c* (Å)	12.4229 (5), 9.6392 (5), 13.2384 (6)
β (°)	102.457 (3)
*V* (Å^3^)	1547.94 (12)
*Z*	4
Radiation type	Cu *K*α
μ (mm^−1^)	0.76
Crystal size (mm)	0.26 × 0.11 × 0.04

Data collection	
Diffractometer	Bruker *SMART* APEXII CCD
Absorption correction	Multi-scan (*SADABS*; Bruker, 2013 ▸)
*T*_min_, *T*_max_	0.599, 0.753
No. of measured, independent and observed [*I* > 2σ(*I*)] reflections	18263, 2895, 2277
*R*_int_	0.037
(sin θ/λ)_max_ (Å^−1^)	0.614

Refinement	
*R*[*F* ^2^ > 2σ(*F* ^2^)], *wR*(*F* ^2^), *S*	0.040, 0.121, 1.05
No. of reflections	2895
No. of parameters	210
H-atom treatment	H atoms treated by a mixture of independent and constrained refinement
Δρ_max_, Δρ_min_ (e Å^−3^)	0.23, −0.14

Computer programs: *APEX2*, *SAINT* (Bruker, 2013 ▸) and *XPREP* (Sheldrick,2008 ▸), *SHELXS97* (Sheldrick, 2008 ▸), *Mercury* (Macrae *et al.*, 2008 ▸), *SHELXL2013* (Sheldrick, 2015 ▸) and *PLATON* (Spek, 2009 ▸).

## supplementary crystallographic information

### Crystal data

**Table:**

C_17_H_17_NO_4_	*F*(000) = 632
*M**_r_* = 299.31	*D*_x_ = 1.284 Mg m^−^^3^
Monoclinic, *P*2_1_/*c*	Cu *K*α radiation, λ = 1.54178 Å
*a* = 12.4229 (5) Å	Cell parameters from 3549 reflections
*b* = 9.6392 (5) Å	θ = 11.5–68.2°
*c* = 13.2384 (6) Å	µ = 0.76 mm^−^^1^
β = 102.457 (3)°	*T* = 296 K
*V* = 1547.94 (12) Å^3^	Block, colorless
*Z* = 4	0.26 × 0.11 × 0.04 mm

### Data collection

**Table:**

Bruker SMART APEXII CCD diffractometer	2277 reflections with *I* > 2σ(*I*)
Radiation source: fine-focus sealed tube	*R*_int_ = 0.037
ω scans	θ_max_ = 71.1°, θ_min_ = 3.6°
Absorption correction: multi-scan (SADABS; Bruker, 2013)	*h* = −15→15
*T*_min_ = 0.599, *T*_max_ = 0.753	*k* = −11→11
18263 measured reflections	*l* = −15→16
2895 independent reflections	

### Refinement

**Table:**

Refinement on *F*^2^	Hydrogen site location: mixed
Least-squares matrix: full	H atoms treated by a mixture of independent and constrained refinement
*R*[*F*^2^ > 2σ(*F*^2^)] = 0.040	*w* = 1/[σ^2^(*F*_o_^2^) + (0.0681*P*)^2^ + 0.1332*P*] where *P* = (*F*_o_^2^ + 2*F*_c_^2^)/3
*wR*(*F*^2^) = 0.121	(Δ/σ)_max_ = 0.001
*S* = 1.05	Δρ_max_ = 0.23 e Å^−^^3^
2895 reflections	Δρ_min_ = −0.14 e Å^−^^3^
210 parameters	Extinction correction: SHELXL2013 (Sheldrick, 2015), Fc^*^=kFc[1+0.001xFc^2^λ^3^/sin(2θ)]^-1/4^
0 restraints	Extinction coefficient: 0.0018 (4)

### Special details

**Table:**

Geometry. All esds (except the esd in the dihedral angle between two l.s. planes) are estimated using the full covariance matrix. The cell esds are taken into account individually in the estimation of esds in distances, angles and torsion angles; correlations between esds in cell parameters are only used when they are defined by crystal symmetry. An approximate (isotropic) treatment of cell esds is used for estimating esds involving l.s. planes.

### Fractional atomic coordinates and isotropic or equivalent isotropic displacement parameters (Å^2^)

**Table:**

	*x*	*y*	*z*	*U*_iso_*/*U*_eq_	
O1	0.40310 (9)	0.79734 (13)	0.24104 (8)	0.0566 (3)	
C1	1.3086 (2)	−0.0296 (3)	0.5552 (2)	0.1030 (8)	
H1A	1.2688	−0.0661	0.4904	0.155*	
H1B	1.3627	−0.0959	0.5878	0.155*	
H1C	1.3447	0.0551	0.5433	0.155*	
C2	1.23138 (15)	−0.00168 (18)	0.62283 (15)	0.0675 (5)	
H2A	1.1963	−0.0870	0.6375	0.081*	
H2B	1.2702	0.0384	0.6877	0.081*	
O3	1.14975 (10)	0.09453 (12)	0.56841 (10)	0.0647 (3)	
C4	1.07204 (13)	0.13648 (19)	0.61646 (13)	0.0577 (4)	
C5	0.99507 (12)	0.23783 (16)	0.55419 (12)	0.0512 (4)	
C6	0.92059 (14)	0.3074 (2)	0.60045 (14)	0.0636 (5)	
H6	0.9217	0.2925	0.6701	0.076*	
C7	0.84532 (14)	0.3980 (2)	0.54458 (14)	0.0609 (4)	
H7	0.7960	0.4438	0.5767	0.073*	
C8	0.84222 (11)	0.42174 (15)	0.44046 (12)	0.0461 (3)	
N1	0.75716 (9)	0.50849 (13)	0.38633 (9)	0.0464 (3)	
C10	0.77038 (12)	0.57994 (15)	0.30896 (12)	0.0472 (3)	
C11	0.68431 (12)	0.66679 (15)	0.24840 (11)	0.0442 (3)	
C12	0.58421 (12)	0.68797 (15)	0.27877 (11)	0.0449 (3)	
H12	0.5723	0.6466	0.3389	0.054*	
C13	0.50380 (11)	0.76950 (14)	0.22013 (11)	0.0416 (3)	
C14	0.52011 (11)	0.83080 (14)	0.12814 (10)	0.0419 (3)	
O15	0.44049 (9)	0.90702 (12)	0.06788 (8)	0.0514 (3)	
C16	0.38242 (16)	0.7484 (3)	0.33611 (15)	0.0777 (6)	
H16A	0.3899	0.6493	0.3391	0.117*	
H16B	0.3089	0.7735	0.3410	0.117*	
H16C	0.4344	0.7893	0.3925	0.117*	
O17	1.06660 (13)	0.09730 (18)	0.70144 (12)	0.0924 (5)	
C18	0.99445 (13)	0.26473 (18)	0.45153 (13)	0.0565 (4)	
H18	1.0455	0.2211	0.4202	0.068*	
C19	0.91880 (13)	0.35567 (18)	0.39485 (12)	0.0545 (4)	
H19	0.9193	0.3726	0.3258	0.065*	
C20	0.70063 (12)	0.73037 (17)	0.15876 (12)	0.0504 (4)	
H20	0.7673	0.7185	0.1387	0.060*	
C21	0.61918 (12)	0.81096 (16)	0.09903 (11)	0.0492 (4)	
H21	0.6312	0.8521	0.0389	0.059*	
H15	0.3862 (18)	0.925 (2)	0.0989 (16)	0.077 (6)*	
H10	0.8383 (16)	0.5785 (18)	0.2855 (14)	0.060 (5)*	

### Atomic displacement parameters (Å^2^)

**Table:**

	*U*^11^	*U*^22^	*U*^33^	*U*^12^	*U*^13^	*U*^23^
O1	0.0488 (6)	0.0743 (7)	0.0522 (6)	0.0172 (5)	0.0227 (5)	0.0181 (5)
C1	0.1014 (17)	0.1064 (18)	0.1052 (18)	0.0560 (15)	0.0308 (14)	0.0244 (15)
C2	0.0615 (10)	0.0546 (9)	0.0794 (12)	0.0128 (8)	−0.0002 (9)	0.0111 (8)
O3	0.0589 (7)	0.0652 (7)	0.0676 (7)	0.0188 (6)	0.0084 (6)	0.0122 (6)
C4	0.0489 (8)	0.0607 (10)	0.0612 (10)	0.0016 (7)	0.0069 (7)	0.0083 (7)
C5	0.0413 (7)	0.0544 (8)	0.0556 (9)	−0.0004 (6)	0.0057 (7)	0.0063 (7)
C6	0.0572 (9)	0.0845 (12)	0.0516 (9)	0.0134 (9)	0.0171 (8)	0.0164 (8)
C7	0.0524 (9)	0.0764 (11)	0.0585 (9)	0.0149 (8)	0.0217 (8)	0.0141 (8)
C8	0.0360 (7)	0.0497 (8)	0.0512 (8)	−0.0022 (6)	0.0067 (6)	0.0026 (6)
N1	0.0388 (6)	0.0501 (7)	0.0490 (7)	0.0018 (5)	0.0061 (5)	0.0021 (5)
C10	0.0381 (7)	0.0510 (8)	0.0520 (8)	−0.0007 (6)	0.0086 (6)	0.0004 (6)
C11	0.0416 (7)	0.0456 (7)	0.0451 (7)	−0.0002 (6)	0.0085 (6)	0.0000 (6)
C12	0.0472 (8)	0.0480 (8)	0.0405 (7)	0.0020 (6)	0.0114 (6)	0.0049 (6)
C13	0.0409 (7)	0.0450 (7)	0.0405 (7)	0.0005 (6)	0.0125 (6)	−0.0003 (6)
C14	0.0439 (7)	0.0435 (7)	0.0376 (7)	−0.0011 (6)	0.0071 (6)	0.0002 (5)
O15	0.0483 (6)	0.0630 (7)	0.0439 (6)	0.0092 (5)	0.0122 (5)	0.0125 (5)
C16	0.0667 (11)	0.1140 (17)	0.0626 (11)	0.0195 (11)	0.0365 (9)	0.0240 (11)
O17	0.0848 (10)	0.1206 (13)	0.0759 (9)	0.0343 (9)	0.0268 (8)	0.0444 (9)
C18	0.0503 (8)	0.0636 (10)	0.0564 (9)	0.0106 (7)	0.0131 (7)	0.0017 (7)
C19	0.0544 (9)	0.0625 (9)	0.0462 (8)	0.0100 (7)	0.0102 (7)	0.0034 (7)
C20	0.0415 (7)	0.0605 (9)	0.0523 (8)	0.0005 (7)	0.0170 (6)	0.0022 (7)
C21	0.0487 (8)	0.0596 (9)	0.0420 (7)	−0.0011 (7)	0.0152 (6)	0.0068 (6)

### Geometric parameters (Å, º)

**Table:**

O1—C13	1.3650 (17)	N1—C10	1.2739 (19)
O1—C16	1.418 (2)	C10—C11	1.455 (2)
C1—C2	1.471 (3)	C10—H10	0.960 (19)
C1—H1A	0.9600	C11—C20	1.389 (2)
C1—H1B	0.9600	C11—C12	1.402 (2)
C1—H1C	0.9600	C12—C13	1.372 (2)
C2—O3	1.446 (2)	C12—H12	0.9300
C2—H2A	0.9700	C13—C14	1.4070 (19)
C2—H2B	0.9700	C14—O15	1.3469 (17)
O3—C4	1.329 (2)	C14—C21	1.380 (2)
C4—O17	1.202 (2)	O15—H15	0.88 (2)
C4—C5	1.486 (2)	C16—H16A	0.9600
C5—C18	1.382 (2)	C16—H16B	0.9600
C5—C6	1.388 (2)	C16—H16C	0.9600
C6—C7	1.373 (2)	C18—C19	1.382 (2)
C6—H6	0.9300	C18—H18	0.9300
C7—C8	1.390 (2)	C19—H19	0.9300
C7—H7	0.9300	C20—C21	1.381 (2)
C8—C19	1.387 (2)	C20—H20	0.9300
C8—N1	1.4152 (18)	C21—H21	0.9300
			
C13—O1—C16	117.64 (12)	C11—C10—H10	115.0 (11)
C2—C1—H1A	109.5	C20—C11—C12	118.87 (13)
C2—C1—H1B	109.5	C20—C11—C10	119.95 (13)
H1A—C1—H1B	109.5	C12—C11—C10	121.18 (13)
C2—C1—H1C	109.5	C13—C12—C11	120.24 (13)
H1A—C1—H1C	109.5	C13—C12—H12	119.9
H1B—C1—H1C	109.5	C11—C12—H12	119.9
O3—C2—C1	107.05 (16)	O1—C13—C12	125.80 (13)
O3—C2—H2A	110.3	O1—C13—C14	113.73 (12)
C1—C2—H2A	110.3	C12—C13—C14	120.46 (13)
O3—C2—H2B	110.3	O15—C14—C21	119.69 (12)
C1—C2—H2B	110.3	O15—C14—C13	121.15 (12)
H2A—C2—H2B	108.6	C21—C14—C13	119.15 (13)
C4—O3—C2	117.42 (14)	C14—O15—H15	111.7 (14)
O17—C4—O3	123.07 (16)	O1—C16—H16A	109.5
O17—C4—C5	124.49 (16)	O1—C16—H16B	109.5
O3—C4—C5	112.43 (14)	H16A—C16—H16B	109.5
C18—C5—C6	118.72 (15)	O1—C16—H16C	109.5
C18—C5—C4	122.37 (15)	H16A—C16—H16C	109.5
C6—C5—C4	118.91 (15)	H16B—C16—H16C	109.5
C7—C6—C5	120.74 (15)	C19—C18—C5	120.78 (15)
C7—C6—H6	119.6	C19—C18—H18	119.6
C5—C6—H6	119.6	C5—C18—H18	119.6
C6—C7—C8	120.60 (15)	C18—C19—C8	120.32 (15)
C6—C7—H7	119.7	C18—C19—H19	119.8
C8—C7—H7	119.7	C8—C19—H19	119.8
C19—C8—C7	118.78 (14)	C21—C20—C11	120.90 (13)
C19—C8—N1	123.94 (14)	C21—C20—H20	119.6
C7—C8—N1	117.23 (13)	C11—C20—H20	119.6
C10—N1—C8	120.99 (12)	C14—C21—C20	120.36 (13)
N1—C10—C11	123.17 (13)	C14—C21—H21	119.8
N1—C10—H10	121.8 (11)	C20—C21—H21	119.8
			
C1—C2—O3—C4	−178.81 (19)	C16—O1—C13—C12	5.9 (2)
C2—O3—C4—O17	−0.7 (3)	C16—O1—C13—C14	−175.42 (16)
C2—O3—C4—C5	178.40 (14)	C11—C12—C13—O1	179.61 (14)
O17—C4—C5—C18	−170.41 (19)	C11—C12—C13—C14	1.1 (2)
O3—C4—C5—C18	10.6 (2)	O1—C13—C14—O15	−1.10 (19)
O17—C4—C5—C6	9.2 (3)	C12—C13—C14—O15	177.62 (13)
O3—C4—C5—C6	−169.87 (16)	O1—C13—C14—C21	179.55 (13)
C18—C5—C6—C7	2.0 (3)	C12—C13—C14—C21	−1.7 (2)
C4—C5—C6—C7	−177.54 (17)	C6—C5—C18—C19	−2.1 (3)
C5—C6—C7—C8	0.0 (3)	C4—C5—C18—C19	177.46 (16)
C6—C7—C8—C19	−2.0 (3)	C5—C18—C19—C8	0.1 (3)
C6—C7—C8—N1	175.45 (16)	C7—C8—C19—C18	1.9 (3)
C19—C8—N1—C10	−31.1 (2)	N1—C8—C19—C18	−175.34 (15)
C7—C8—N1—C10	151.56 (15)	C12—C11—C20—C21	−1.3 (2)
C8—N1—C10—C11	177.71 (13)	C10—C11—C20—C21	178.69 (14)
N1—C10—C11—C20	−173.29 (14)	O15—C14—C21—C20	−178.47 (14)
N1—C10—C11—C12	6.7 (2)	C13—C14—C21—C20	0.9 (2)
C20—C11—C12—C13	0.5 (2)	C11—C20—C21—C14	0.6 (2)
C10—C11—C12—C13	−179.54 (13)		

### Hydrogen-bond geometry (Å, º)

Cg2 is the centroid of the C11–C14/C20/C21 ring.

**Table:**

*D*—H···*A*	*D*—H	H···*A*	*D*···*A*	*D*—H···*A*
O15—H15···N1^i^	0.88 (2)	2.00 (2)	2.828 (2)	156 (2)
C2—H2*B*···*Cg*2^ii^	0.97	2.87	3.766 (2)	154

Symmetry codes: (i) −*x*+1, *y*+1/2, −*z*+1/2; (ii) −*x*+2, −*y*+1, −*z*+1.
