# Supplementary material for: Crystal structure of ethyl 4-[(E)-(4-hy­droxy-3-meth­oxy­benzyl­idene)amino]­benzoate: a p-hy­droxy Schiff base
Source: Acta Crystallogr E Crystallogr Commun. 2016 Jun 14;72(Pt 7):951–4. doi: 10.1107/S2056989016008999 (PMC4992913; doi:10.1107/S2056989016008999)
Supplement: Supplementary file 3 [file e-72-00951-sup3.pdf]

Supporting information

S1.

A single crystal of $C_{17}H_{17}NO_4$ [BZCVAN] was selected and mounted onto a goniometer. The crystal was maintained at 296 (2) K during data collection. X-ray diffraction data for BZCVAN were collected using a Bruker-AXS SMART-APEXII CCD diffractometer using Kα radiation (λ = 1.54178 Å). Indexing was performed using APEX2 (Bruker., 2013) (Difference Vectors method). Data integration and reduction were performed using SaintPlus 6.01 (Bruker., 2013). Absorption correction was performed by multi-scan method implemented in SADABS (Sheldrick G. M., 1996). Space groups were determined using XPREP implemented in APEX2. The structure was solved using SHELXS-97 (direct methods) and refined using SHELXL-2013 (Sheldrick, G. M., 2008) (full-matrix least-squares on F^2) contained in APEX2 (Bruker., 2013., Sheldrick, G. M., 2008), WinGX v1.70.01 (Farrugia L. J., 1999., Sheldrick G. M., 1990., 1997., 2008) and OLEX2 (Sheldrick, G. M. (2008)., Dolomanov O. V., Bourhis, L. J., Gildea R. J., Howard, J. A. K., Puschmann, H., 2009). All non-hydrogen atoms were refined anisotropically. . Hydrogen atom of hydroxyl group and atom H10 have been found from difference Fourier map and were freely refined. The remaining hydrogen atoms were placed in geometrically calculated positions and included in the refinement process using riding model with isotropic thermal parameters: Uiso(H) = 1.2Ueq(-CH,-CH_2) and Uiso(H) = 1.5Ueq(-CH_3). Crystal data and refinement conditions are shown in Table 1.

Table S1

Fractional Atomic Coordinates (×10^4) and Equivalent Isotropic Displacement Parameters (Å^2×10^3). U_{eq} is defined as 1/3 of the trace of the orthogonalised U_{IJ} tensor.

Atom	*x*	*y*	*z*	U(eq)
O1	4031.0 (9)	7973.4 (1)	2410.4 (8)	56.6 (3)
C1	13086 (2)	-296 (3)	5553 (2)	103.0 (8)
C2	12313.7 (1)	-16.8 (2)	6228.4 (1)	67.4 (5)
O3	11497.5 (1)	945.2 (1)	5683.9 (1)	64.7 (3)
C4	10720.4 (1)	1364.9 (2)	6164.5 (1)	57.7 (4)
C5	9950.7 (1)	2378.3 (2)	5541.9 (1)	51.1 (4)
C6	9206.1 (1)	3074 (2)	6004.4 (1)	63.6 (5)
C7	8453.1 (1)	3980 (2)	5445.6 (1)	60.9 (4)
C8	8422.3 (1)	4217.5 (2)	4404.7 (1)	46.0 (3)

N1	7571.6 (9)	5084.9 (1)	3863.4 (9)	46.4 (3)
C10	7703.1 (1)	5799.7 (1)	3089.2 (1)	47.3 (4)
C11	6843.1 (1)	6667.8 (1)	2484.0 (1)	44.2 (3)
C12	5842.0 (1)	6879.6 (1)	2787.7 (1)	44.9 (3)
C13	5038.0 (1)	7694.9 (1)	2201.2 (1)	41.5 (3)
C14	5201.2 (1)	8307.8 (1)	1281.5 (1)	41.9 (3)
O15	4404.9 (9)	9070.2 (1)	678.8 (8)	51.4 (3)
C16	3824.3 (2)	7484 (3)	3361.1 (1)	77.6 (6)
O17	10666.2 (1)	973.1 (2)	7014.6 (1)	92.2 (5)
C18	9944.6 (1)	2647.3 (2)	4515.4 (1)	56.5 (4)
C19	9188.1 (1)	3556.8 (2)	3948.6 (1)	54.4 (4)
C20	7006.3 (1)	7303.6 (2)	1587.6 (1)	50.3 (4)
C21	6191.8 (1)	8109.6 (2)	990.5 (1)	49.2(4)

Table S2

Torsion Angles for BZCVAN.

A	B	C	D	Angle/°	A	B	C	D	Angle/°
O1	C13	C14	O15	-1.11 (2)	N1	C10	C11	C20	-173.3 (1)
O1	C13	C14	C21	179.5 (1)	C10	C11	C12	C13	-179.5 (1)
C1	C2	O3	C4	-178.8 (2)	C10	C11	C20	C21	178.7 (1)
C2	O3	C4	C5	178.4 (1)	C11	C12	C13	O1	179.6 (1)
C2	O3	C4	O17	-0.7 (3)	C11	C12	C13	C14	1.1 (2)
O3	C4	C5	C6	-169.9 (2)	C11	C20	C21	C14	0.6 (2)
O3	C4	C5	C18	10.5 (2)	C12	C11	C20	C21	-1.3 (2)
C4	C5	C18	C19	177.5 (2)	C12	C13	C14	C21	-1.7 (2)
C5	C6	C7	C8	0.0 (3)	C13	C14	C21	C20	0.9 (2)
C5	C18	C19	C8	0.1 (3)	O15	C14	C21	C20	-178.5 (1)
C6	C5	C18	C19	-2.1 (3)	C16	O1	C13	C12	5.9 (2)
C6	C7	C8	N1	175.5 (2)	C16	O1	C13	C14	-175.4 (2)
C6	C7	C8	C19	-2.0 (3)	O17	C4	C5	C6	9.2 (3)
C7	C8	N1	C10	151.6 (1)	O17	C4	C5	C18	-170.4 (2)
C7	C8	C19	C18	1.9 (3)	C18	C5	C6	C7	2.1 (3)
C8	N1	C10	C11	177.7 (1)	C19	C8	N1	C10	-31.1 (2)
N1	C8	C19	C18	-175.3 (1)	C20	C11	C12	C13	0.4 (2)
N1	C10	C11	C12	6.7 (2)					

Table S3

Hydrogen Atom Coordinates (Å×10^4) and Isotropic Displacement Parameters (Å^2×10^3) for BZCVAN.

**Atom**	***x***	***y***	***z***	**U(eq)**
H1A	12689	-661	4904	154
H1B	13627	-959	5879	154
H1C	13447	550	5433	154
H2A	11963	-870	6376	81
H2B	12701	384	6878	81
H6	9217	2926	6701	76
H7	7959	4438	5767	73
H10	8382	5764	2902	57
H12	5722	6466	3389	54
H16A	3899	6493	3391	116
H16B	3090	7735	3411	116
H16C	4344	7893	3925	116
H18	10456	2211	4202	68
H19	9193	3726	3258	65
H20	7673	7185	1387	60
H21	6312	8521	389	59
H15	3858 (18)	9250 (20)	983 (16)	76 (6)

1

Comparison of experimental and the calculated PXRD of BZCVAN.

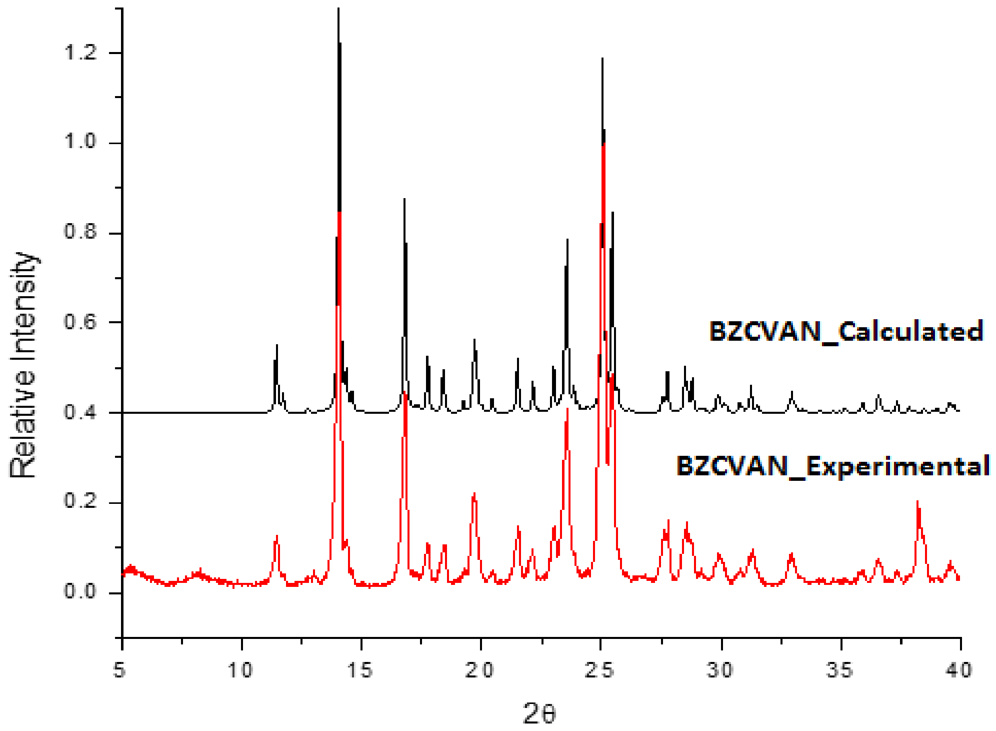

Figure S1

DSC of BZCVAN

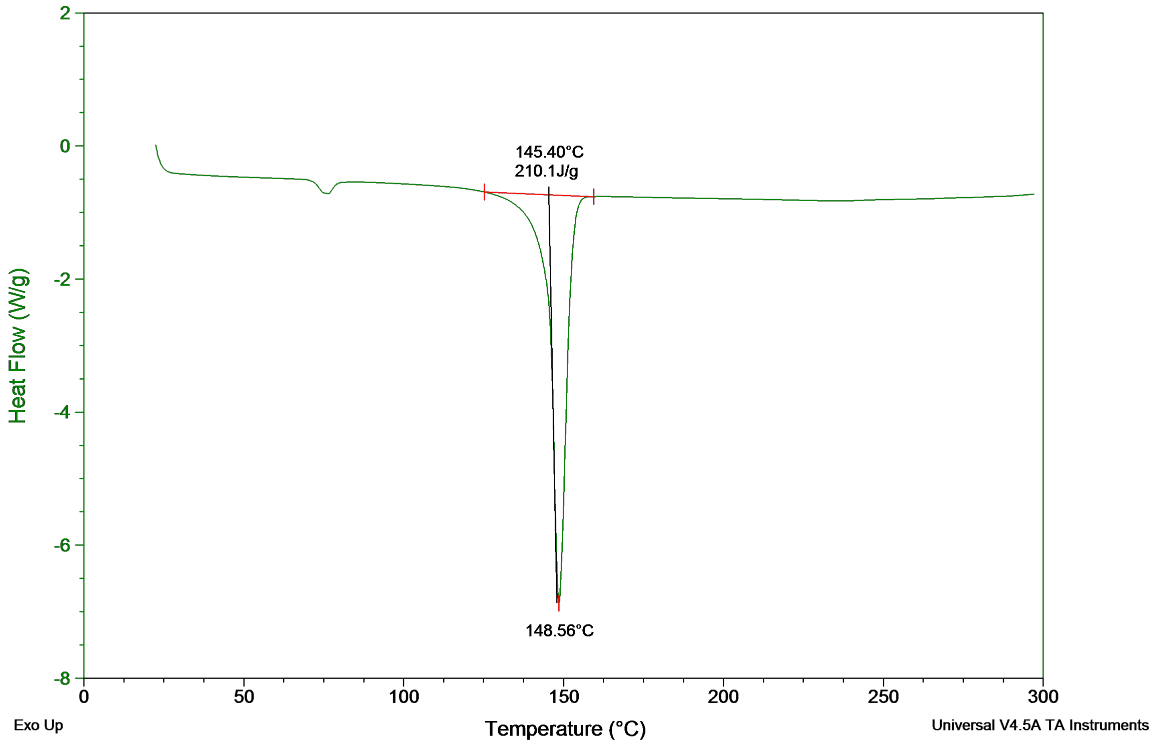

145.40°C
210.1J/g
148.56°C
Heat Flow (W/g)
2
0
-2
-4
-6
-8
0
50
100
150
200
250
300
Exo Up
Temperature (°C)
Universal V4.5A TA Instruments
